# Species Identification, Insecticide Resistance and TYLCV Detection of *Bemisia tabaci* in Kashgar, Xinjiang

**DOI:** 10.3390/insects17010112

**Published:** 2026-01-20

**Authors:** Weina Gu, Jing Yang, Qi Li, Jinyu Hu, Rong Zhang, Shaoli Wang, Youjun Zhang, Qi Su, Xin Yang

**Affiliations:** 1 College of Agriculture, Yangtze University, Jinzhou 434020, China; weinagu_2022@163.com (W.G.); sp141611@163.com (R.Z.); 2 State Key Laboratory of Vegetable Biobreeding, Department of Plant Protection, Institute of Vegetables and Flowers, Chinese Academy of Agricultural Sciences, Beijing 100081, China; yangjing1185@163.com (J.Y.); liqi20001002@163.com (Q.L.); jinyuhu0623@163.com (J.H.); wangshaoli@caas.cn (S.W.); zhangyoujun@caas.cn (Y.Z.)

**Keywords:** *B. tabaci*, cotton, tomato, insecticide resistance, TYLCV

## Abstract

The rapid evolution of insecticide resistance in *Bemisia tabaci* (Gennadius) (Hemiptera: Aleyrodidae) threatens effective pest management in key crops. This study characterized *B*. *tabaci* populations from cotton and tomato fields in Kashgar (September–October 2024) using mtCOI-RFLP for cryptic species identification, leaf-dip bioassays with 13 insecticides, and PCR detection of tomato yellow leaf curl virus (TYLCV). All analyzed individuals belonged to the Mediterranean (MED) cryptic species. Extreme resistance was observed to imidacloprid (RR = 320.65) and pyridaben (RR = 331.29), while nitenpyram (RR = 1.77) and the emamectin benzoate–chlorantraniliprole mixture (RR = 2.13) remained effective. TYLCV was detected in 97.5% of adults from tomato greenhouses. These findings provide a concise assessment of resistance status, species identification, and virus prevalence in *B*. *tabaci*, informing sustainable management strategies in cotton and tomato production.

## Simple Summary

Rapid development of insecticide resistance in *Bemisia tabaci* poses serious threats to key crops such as tomato and cotton. In this study, populations collected from cotton and tomato fields in Kashgar in 2024 were examined for species identification, insecticide susceptibility, and tomato yellow leaf curl virus (TYLCV) infection. Molecular identification revealed that all successfully tested individuals belonged to the Mediterranean (MED) cryptic species. Bioassays with 13 insecticides showed that neonicotinoids, including imidacloprid, had lost effectiveness due to high resistance levels, whereas nitenpyram and the emamectin benzoate–chlorantraniliprole mixture retained strong efficacy. Approximately 98% of the *Bemisia tabaci* individuals collected from tomato plants were positive for TYLCV, indicating a high potential for virus transmission within tomato crops. These findings provide critical insights into the resistance status, species identification, and virus-carrying capacity of *B*. *tabaci* in Kashgar, offering guidance for sustainable pest management strategies in key crops.

## 1. Introduction

*Bemisia tabaci* is a globally distributed agricultural pest of critical importance with its severe damage necessitating urgent control measures [1]. Both adults and nymphs feed on plant sap by piercing–sucking, resulting in leaf chlorosis [2], yellowing, wilting, and ultimately plant mortality; simultaneously, they excrete copious honeydew that induces sooty mold formation, impairs photosynthetic efficiency, and contaminates crops (e.g., in-creasing sugar content in cotton fibers, disrupting textile processing) [3]. Furthermore, *B. tabaci* serves as an efficient vector for numerous plant viruses [4], transmitting over 100 viral species [5] (e.g., criniviruses [2] and tomato yellow leaf curl virus (TYLCV) [6]), leading to significant yield losses and quality degradation. Driven by the expansion of green-house cultivation in temperate regions [3], its damage range has expanded from tropical/subtropical zones to a global scale [7]. Over the past two decades, epidemic outbreaks of *B. tabaci* have occurred worldwide, posing severe threats to cash crops such as cotton, vegetables, and ornamentals [8]. Consequently, enhancing *B. tabaci* management is imperative for securing agricultural safety.

However, the control of *B. tabaci* faces two core challenges: complex biotypes (or cryptic species) with high invasiveness [9], and strong and evolving insecticide resistance [10]. In terms of species complexity, *B. tabaci* is not a single species but a complex of at least 24 morphologically similar yet genetically distinct species [10]. Eleven high-level groups (e.g., MED and MEAM1 cryptic species) have been identified to date [7]. This complexity drives high invasiveness: invasive cryptic species like MEAM1 (also known as B biotype) and MED (also known as Q biotype) competitively displace native populations (e.g., ZHJ-type) [7,11], expanding globally, and enhancing virus transmission capability (e.g., MED spread from southeastern to northern China, with rising imidacloprid resistance gene frequencies) [12,13]. For instance, high resistance levels have been observed in *B. tabaci* populations from Beijing, Shandong, and Hunan [9]. Since 1996, the Mediterranean (MED) cryptic species *B. tabaci* in Almería, Spain, has exhibited over 100-fold resistance to imidacloprid, thiamethoxam, and acetamiprid, with resistance stably maintained without selection pressure [14]. In US greenhouse and open-field crop systems, imidacloprid resistance in the Mediterranean (MED) cryptic species *B. tabaci* has significantly increased, sharing similar mechanisms (P450-mediated metabolic enhancement) with Spanish populations [15]. Regarding insecticide resistance, *B. tabaci* has developed significant resistance to common insecticides (e.g., imidacloprid and abamectin) [16], primarily due to the overexpression of cytochrome P450 genes (e.g., *CYP4V2* and *CYP6CXI*) [9]. Recent studies indicate that *B. tabaci* may acquire the plant-derived detoxification gene *BtPMaT1* through horizontal gene transfer (HGT), enabling metabolism of phenolic glycoside toxins [17]. In Xinjiang populations of China, the A58T/R79E double mutation in the nAChR β1 subunit has been identified, altering receptor charge distribution to confer target-site resistance, which inactivates seven neonicotinoid agents (e.g., thiamethoxam and acetamiprid) with resistance ratio [18]. Long-term misuse of chemical pesticides accelerates the evolution of resistance mechanisms [19].

Southern Xinjiang, in particular, is characterized by extensive contiguous green-house complexes [20]. Bordering multiple Central Asian nations and adopting a staggered cropping system with sequential crop maturation [21], this region provides a continuous and stable host habitat for *B. tabaci* [22]. Moreover, the oasis-desert transition zones in Xinjiang (e.g., Kashgar at 39.5° N) feature a mosaic of enclosed (greenhouse) and open (field) agricultural habitats shaped by irrigated farming, providing year-round breeding conditions for *B. tabaci* [23]. Thus, systematic surveys in Kashgar will not only elucidate local population dynamics and resistance mechanisms but also offer a scalable reference framework for global *B. tabaci* management. This study aims to analyze the distribution of cryptic species, insecticide resistance levels, and viral carriage status of *B. tabaci* through field investigations in Kashgar, Xinjiang, to develop more effective integrated pest management strategies. We anticipate that the findings will contribute to sustainable management and enhanced agricultural biosecurity.

## 2. Materials and Methods

### 2.1. Field Populations

*Bemisia tabaci* populations analyzed in this study were collected from Jiashi County (37°55′–39°16′ N, 76°20′–78°00′ E), Kashgar Prefecture, Xinjiang Uygur Autonomous Region, China. All populations originated from the same county-level geographic area and therefore shared identical GPS coordinates. Sampling was conducted within a consistent time window between September and October 2024 to minimize potential temporal variation among populations. A total of five field populations were sampled, including one population collected from open-field cotton and four populations collected from tomato greenhouses, with cotton and tomato serving as the respective host plants.

**Figure 1:**
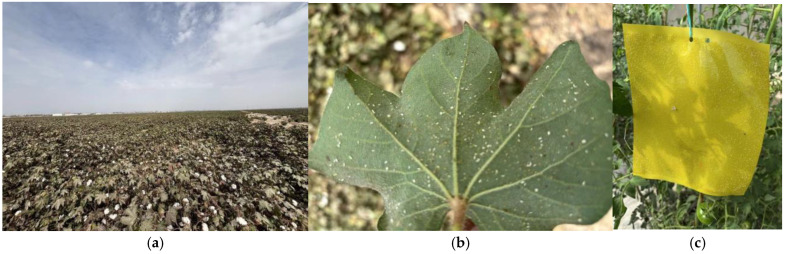
(**a**) (Cotton field): Abscisic acid (ABA) spraying during cotton harvesting induced leaf senescence, resulting in extensive chlorosis and defoliation; (**b**) (Leaf microhabitat): High-density aggregation of *B. tabaci* populations (adults + nymphs + eggs > 1000 per leaf) was observed on cotton leaves; (**c**) (Facility tomato greenhouse): Yellow sticky traps in greenhouse tomato systems recorded > 10,000 adult captures per trap.

Xinjiang is a major agricultural region in China and the country’s primary production area for processing tomatoes, contributing more than 80% of the national output. Kashgar Prefecture represents an important production area in southern Xinjiang, benefiting from favorable agroclimatic conditions, including high annual sunshine duration (>2800 h) and a large diurnal temperature range (ΔT ≥ 15 °C). In addition, Xinjiang accounts for over 90% of China’s total cotton production, with Kashgar contributing approximately 25% of the region’s cotton yield. Meanwhile, *B*. *tabaci* has emerged as an important pest in Xinjiang and is commonly detected in tomato and cotton cropping systems, as illustrated in Figure 1.

### 2.2. Total DNA Extraction from B. tabaci

Fifty adult *B. tabaci* individuals were randomly selected for genomic DNA extraction. Genomic DNA was obtained from single adults using the Kapa Express Extract DNA Extraction Kit (PuKaiRui Biotechnology Co., Ltd., Beijing, China). For each sample, the extraction mixture was prepared by combining 26.5 µL ddH_2_O, 3.0 µL buffer, and 0.5 µL enzyme solution. An individual whitefly was placed into a 1.5 mL microcentrifuge tube, after which grinding beads and the prepared extraction solution were added. Samples were homogenized using a bead mill until complete tissue disruption was achieved. The homogenate was then briefly centrifuged at 8000 rpm for 30 s, and the resulting supernatant was transferred to a sterile PCR tube. Enzymatic reactions were performed in a PCR thermocycler with incubation at 75 °C for 10 min to allow protein digestion, followed by heat inactivation at 95 °C for 5 min.

### 2.3. Identification of B. tabaci Species

Species identification of adult *Bemisia tabaci* was conducted using a restriction fragment length polymorphism (RFLP) assay based on sequence polymorphisms in the mitochondrial cytochrome oxidase I (mtCOI) gene that distinguish the MED and MEAM1 species. A total of 20 adult individuals from the cotton population were analyzed for species identification. A 620 bp fragment of mtCOI was amplified using 2× Es Taq Master Mix (Beijing ComWin Biotech Co., Ltd., Beijing, China) with the primer pair Cl-J-2195 and R-BQ-2819. PCR reactions were performed in a total volume of 20.0 µL containing PCR master mix, primers, genomic DNA template, and ddH_2_O. Thermal cycling consisted of an initial denaturation at 94 °C for 2 min, followed by 30 cycles of denaturation at 94 °C for 30 s, annealing at 55 °C for 30 s, and extension at 72 °C for 30 s, with a final extension at 72 °C for 2 min.

PCR products were digested with ASEI restriction endonuclease (Blinc Biotechnology Co., Ltd., Beijing, China) and separated by electrophoresis on a 1.5% agarose gel. Fragment patterns were visualized using a Tanon MINI Space 3000 gel documentation system (Tanon Life Science, Shanghai, China) to distinguish MED and MEAM1 species.

### 2.4. Insecticides

All insecticides used in this study are listed in Table 1, covering three major mechanistic classes: Antibiotics: Abamectin, a macrocyclic lactone neurotoxin, induces insect paralysis by activating glutamate-gated chloride channels [24]; Neonicotinoids: Including imidacloprid and thiamethoxam, these compounds exert systemic efficacy via selective agonism of nicotinic acetylcholine receptors (nAChRs). Thiacloprid is prioritized in ecologically sensitive areas due to its low bee toxicity (contact LD_50_ = 17.3 μg/bee) [25]; Pyrethroids: Bifenthrin and deltamethrin cause neuronal hyperexcitation by modulating voltage-gated sodium channels [26]. Additionally, flonicamid acts as a selective feeding blocker by inhibiting stylet muscle contraction in aphids [27], while pyridaben suppresses mitochondrial complex I to eradicate mites [28]. The emamectin–chlorantraniliprole coformulation synergistically targets neural pathways and calcium channels for lepidopteran control [29].

### 2.5. Adult Bioassays with the Thirteen Pesticides

Adult insecticide resistance bioassays were conducted using 13 insecticides representing different chemical classes on a *B*. *tabaci* population collected from open-field cotton plots. Bioassays were performed following the leaf-dip method described by Zheng et al. [30], with minor modifications. For each insecticide, six serial concentrations were prepared based on field-recommended application rates using a stepwise dilution scheme. Fresh, untreated cotton leaves were punched into 22 mm discs and immersed in the test solutions for 15 s. Three biological replicates were prepared per concentration, and control discs were dipped in distilled water. After air-drying on filter paper, leaf discs were placed on 1.5% agar in glass bioassay tubes, with the abaxial surface facing upward. Approximately 20 adult whiteflies were introduced into each tube using an aspirator, with three tubes per concentration. Bioassays were maintained under controlled conditions (26 ± 1 °C, 16 h:8 h light/dark photoperiod). Adult mortality was recorded after 48 h, and percentage mortality was calculated accordingly.

### 2.6. TYLCV Detection of B. tabaci

Tomato yellow leaf curl virus (TYLCV, GenBank accession No. NC_004005) was the target virus examined in this study. A total of 81 adult *B. tabaci* individuals collected from tomato greenhouses were analyzed for TYLCV detection. Genomic DNA extracted from adult individual whiteflies was used as the template for PCR-based detection. Each PCR reaction was conducted in a total volume of 20.0 µL, consisting of 10.0 µL PCR mix, 7.0 µL ddH_2_O, 1.0 µL of each primer, and 1.0 µL DNA template. TYLCV-specific primers TYLCV-61 (5′-ATACTTGGACACCTAATGGC-3′) and TYLCV-473 (5′-AGTCACGGGCCCTTACAA-3′) were used for amplification. PCR amplification was performed with an initial denaturation at 94 °C for 2 min, followed by 32 cycles of denaturation at 94 °C for 30 s, annealing at 60 °C for 30 s, and extension at 72 °C for 30 s, and a final extension step at 72 °C for 2 min. Amplified products were directly resolved by agarose gel electrophoresis, producing a diagnostic band of 412 bp. Samples lacking this specific band were considered negative for TYLCV infection.

### 2.7. Statistical Analysis

A pesticide-susceptible reference strain of *B. tabaci* (designated THS) was established in 2008 and has since been maintained under insecticide-free conditions. This strain serves as the baseline for bioassays to monitor resistance evolution and ensure experimental consistency. Field-collected populations were evaluated alongside untreated controls to account for natural mortality. Bioassay data were processed using POLO Plus 2.0 to derive median lethal concentrations (LC_50_). Resistance ratios (RR) were calculated as: RR = LC_50_ (field population)/LC_50_ (THS reference strain). Published sensitivity baselines were referenced for resistance assessment: pyridaben (0.52 mg L^−1^) [30], emamectin benzoate (2.92 mg L^−1^) [30], spinetoram (1.38 mg L^−1^) [31], nitenpyram (2.68 mg L^−1^) [32], flupyradifurone (18.63 mg L^−1^) [32], dinotefuran (6.01 mg L^−1^) [33], flonicamid (7.52 mg L^−1^) [34], thiameth-oxam (1.19 mg L^−1^) [35], imidacloprid (0.99 mg L^−1^) [35], abamectin (0.06 mg L^−1^) [36], and acetamiprid (1.80 mg L^−1^) [36]. Resistance levels were categorized according to IRAC guidelines: very high (RR > 100), high (RR = 31–100), moderate (RR = 11–30), low (RR = 1–10), and susceptible (RR < 1) [37].

## 3. Results

### 3.1. Species Determination of the Cotton-Collected B. tabaci Population

**Figure 2:**
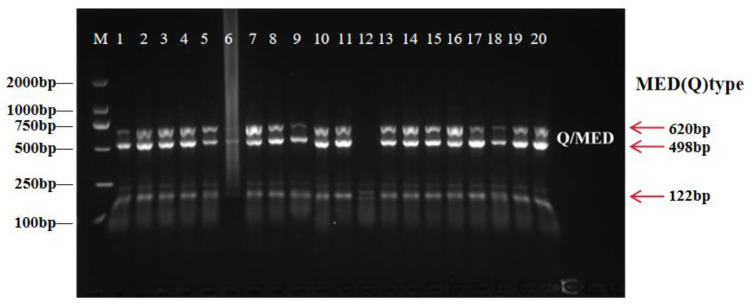
Identification of *B. tabaci* species. M: marker D2000.

Molecular identification based on mtCOI-RFLP analysis indicated that the *B*. *tabaci* population collected from open-field cotton in Kashgar, Xinjiang belonged predominantly to the MED species. Among the 20 adult individuals examined, 19 exhibited the characteristic MED-specific ASEI digestion pattern, producing two fragments of 498 bp and 122 bp, whereas one individual did not yield a clear digestion pattern. No undigested 620 bp fragment corresponding to the MEAM1 species was detected in any of the analyzed samples (Figure 2). Thus, excluding the one unclear sample, all successfully analyzed individuals (19/19) were confirmed as MED, corresponding to 100% of the clearly identified samples.

### 3.2. Adult Resistance to Thirteen Insecticides in the Cotton-Collected MED Population

**Figure 3:**
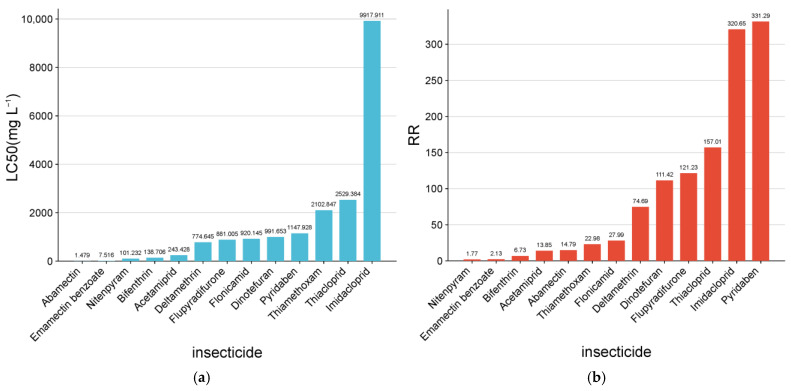
Insecticide resistance of the *Bemisia tabaci* population collected from open-field cotton in Kashgar, Xinjiang, China. (**a**) Histogram showing the median lethal concentrations (LC_50_) of 13 insecticides; the x-axis is arranged from high to low LC_50_. (**b**) Bar chart showing the resistance factors (Resistance Ratio) of the same population; the x-axis is arranged from small to large resistance factors.

Bioassay results revealed that the MED adult *B. tabaci* population from open-field cotton in Kashgar had developed widespread and variable resistance to multiple insecticides (Table 2; Figure 3). Extreme resistance (RR > 300) was detected to imidacloprid (RR = 320.65) and pyridaben (RR = 331.29). High resistance (RR > 100) was observed for thiacloprid (RR = 157.01), dinotefuran (RR = 111.42), and flupyradifurone (RR = 121.23). Moderate to high resistance (RR > 10) was recorded for thiamethoxam, acetamiprid (RR = 13.85), deltamethrin (RR = 74.69), and flonicamid (RR = 27.99).

By contrast, relatively low resistance levels were observed for nitenpyram (RR = 1.77), bifenthrin (RR = 6.73), and abamectin-based insecticides. Although abamectin exhibited a moderate resistance ratio (RR = 14.79), its low LC_50_ value (1.479 mg L^−1^) indicated retained high intrinsic toxicity. Notably, the emamectin benzoate–chlorantraniliprole mixture showed the highest efficacy, with the lowest resistance ratio (RR = 2.13; LC_50_ = 7.516 mg L^−1^), suggesting strong control potential against this population.

### 3.3. TYLCV Infection in Cotton- and Tomato-Collected B. tabaci Populations

**Figure 4:**
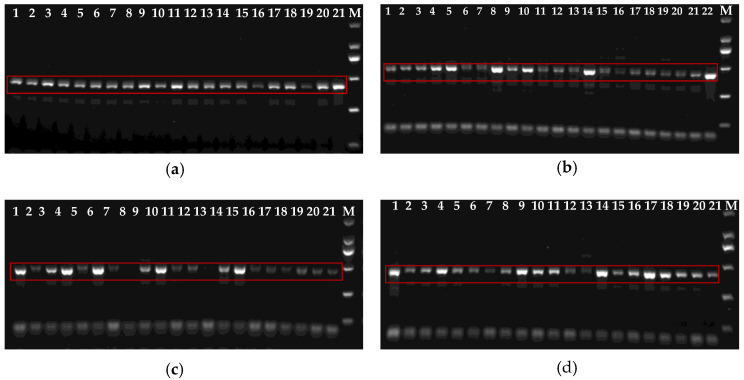
Detection of TYLCV in *B. tabaci* collected from four tomato populations. Panels (**a**–**d**) represent TYLCV detection results for each tomato population, respectively. The last lane in each panel corresponds to the TYLCV-positive control. M, Marker; from top to bottom, the DNA ladder sizes are 2000, 1000, 750, 500, 250, and 100 bp. The red box indicates the TYLCV band at 412 bp.

TYLCV detection was conducted on adult *B. tabaci* individuals collected from four tomato greenhouse populations. Among the 81 individuals tested, 79 were positive for TYLCV, resulting in an overall infection rate of 97.5% (Figure 4). These results indicate a high prevalence of TYLCV infection in *B. tabaci* populations from tomato greenhouses in the surveyed region.

## 4. Discussion

Our mtCOI-RFLP analysis of *B. tabaci* from open-field cotton in Kashgar revealed a population overwhelmingly dominated by MED, with all 19 adults analyzed (100%) exhibiting the MED-specific ASEI digestion pattern and no MEAM1 detected. This indicates a clear dominance of MED in the surveyed fields. Notably, the displacement of indigenous *B. tabaci* biotypes/species by MED or MEAM1 appears to be a widespread phenomenon in China. Similar patterns have been reported in other regions of China. Jia et al. [38] observed predominantly Q-type populations with only minor B-type occurrence in other regions, suggesting that local ecological conditions, host availability, and historical insecticide usage may shape species composition. Other studies confirm that invasive MED and MEAM1 have largely replaced native cryptic species across multiple regions: in Henan, invasive MED and MEAM1 predominated while native cryptic species remained at low prevalence [39], and in eastern China, biotype Q (MED) rapidly replaced biotype B [40]. These observations are consistent with global reports of MED expansion, particularly in regions with intensive neonicotinoid use, such as Spain, Italy [41], and Israel [14].

Long-term neonicotinoid selection pressure likely contributes to MED dominance in Kashgar. Genetic analyses indicate that MED possesses higher diversity and adaptability, while MEAM1 suffers from reduced fitness due to bottlenecks [42]. Reproductive isolation further limits gene flow, and MED can outcompete MEAM1 by reducing its feeding efficiency, partly through plant-mediated defense mechanisms such as jasmonic acid signaling [43,44,45]. It should be noted that cryptic species identification was limited to cotton-collected *B*. *tabaci*, and tomato-collected populations were not analyzed. Future studies should extend species identification to other host plants to fully understand the distribution and dynamics of *B*. *tabaci* cryptic species in the region.

Previous studies demonstrated low LC_50_ values (<10 ppm for most compounds) in the reference *B. tabaci* strain across all 13 insecticides assessed [46]. Resistance monitoring of the Mediterranean (MED) *B. tabaci* cryptic species in Kashgar, Xinjiang revealed widespread high resistance to conventional insecticides, with neonicotinoids exhibiting the most severe resistance. Thiamethoxam, thiacloprid, dinotefuran, acetamiprid and imidacloprid showed extreme resistance levels (e.g., imidacloprid RR = 320.65, LC_50_ = 9917.911 ppm), while flupyradifurone, a recently introduced neonicotinoid, rapidly elicited high resistance. This aligns with global neonicotinoid resistance trends, primarily attributed to enhanced metabolic detoxification (e.g., cytochrome P450 overexpression) induced by long-term sole insecticide usage [47]. Consistent with previous research reporting P450-mediated metabolic enhancement in MED [48], another study further identified target-site resistance via nAChR β1 subunit A58V mutation in the Ürümqi population [49], suggesting potential parallel mechanisms in the Kashgar MED population.

Notably, nitenpyram (RR = 1.77) and the pyrethroid bifenthrin (RR = 6.73) demon-strated low resistance, serving as viable rotation candidates [50], whereas the combination insecticide emamectin–chlorantraniliprole (RR = 2.13) exhibited optimal control efficacy, validating mixture strategies for resistance mitigation [51]. Although abamectin (RR = 14.79) showed moderate resistance, its potential accumulation risk warrants vigilance. Critically, high flupyradifurone resistance in Kashgar (similar to Florida MED populations) warns of rapid efficacy loss in novel insecticides, demanding global vigilance against resistance dissemination. While sulfoxaflor (sulfoximine class) was not tested here, previous studies indicate it lacks cross-resistance with neonicotinoids [52], suggesting it as a promising alternative for integrated management. However, reliance solely on bioassays in this study precluded quantification of underlying metabolic (e.g., P450 activity) or target-site (e.g., nAChR mutations) contributions. We therefore recommend integrated analyses combining metabolic enzyme assays, target-gene mutation screening, and RNAi validation to identify primary resistance mechanisms in the Kashgar MED population, thereby informing more precise and sustainable insecticide deployment strategies.

TYLCV is widely carried in *B. tabaci* populations, reflecting its high transmission potential and epidemic capacity. In our study, TYLCV was detected in 97.5% of adults (79/81) collected from four tomato greenhouse populations in Kashgar (Figure 4), confirming a high prevalence in the region. Given that these whiteflies were collected directly from TYLCV-infected tomato plants, the extremely high viruliferous rate indicates efficient virus acquisition by *B. tabaci* under greenhouse conditions. Such viruliferous adults can act as persistent sources of inoculum, facilitating rapid secondary spread of TYLCV within and between greenhouses. Moreover, continuous exposure to infected host plants may enhance the likelihood of repeated virus acquisition, thereby sustaining high infection pressure in local cropping systems. It should be noted that TYLCV analyses were restricted to adult *Bemisia tabaci* collected from tomato plants, as cotton-collected populations were not subjected to TYLCV detection in this study.

Previous studies have shown that efficient retention of viral particles in the insect’s salivary glands, suppression of host jasmonic acid (JA)-dependent defenses, and increased vector fecundity collectively facilitate rapid viral spread [53]. This virus-vector mutualism confers absolute dominance to viruliferous individuals within the population. Concurrently, the arid climate (high temperature, low humidity) in Kashgar promotes whitefly reproduction and viral accumulation [54], while extensively cultivated susceptible hosts (e.g., tomato, cotton) provide a foundation for TYLCV epidemics [55]. The transovarial transmission of TYLCV results in innate viral carriage in offspring [56]. Furthermore, volatile organic compounds (VOCs) released by infected plants attract whiteflies for feeding [45], establishing an “infection–aggregation” positive feedback loop. Collectively, these mechanisms, together with our field observations, suggest that environmental and host factors likely contribute to the high TYLCV prevalence observed in Kashgar *B*. *tabaci* populations.

## 5. Conclusions

Our study demonstrates the dominance of the Mediterranean (MED) cryptic species of *Bemisia tabaci* in cotton fields in Kashgar, Xinjiang. The high levels of resistance to multiple neonicotinoids highlight the urgent need for strategic insecticide rotation and targeted control measures. The widespread prevalence of TYLCV in field populations underscores the potential for rapid viral spread. Collectively, these findings provide an integrated understanding of cryptic species composition, insecticide resistance, and virus dynamics in the region, offering a solid scientific foundation for developing effective, region-specific integrated pest management (IPM) programs to maintain crop productivity and ensure biosecurity.

## Figures and Tables

**Table 1:** Background information on the insecticides used in this study.

Insecticide	Formulation	Class of Insecticide	Active Ingredient	Manufacturer	Recommended Field Concentration (mg L^−1^)
Abamectin	Emulsifiable Concentrate (EC)	Biological	1.8%	Chemical Industry Co., Ltd. (Tokyo, Japan)	10.8–14.4
Thiamethoxam	Water-Dispersible Granule (WG)	Neonicotinoid	25%	Syngenta (Basel, Switzerland)	35–75
Flupyradifurone	Soluble Concentrate (SL)	Neonicotinoid	17%	Bayer (China) Limited (Beijing, China)	333–666
Imidacloprid	Water-Dispersible Granule (WG)	Neonicotinoid	70%	Bayer (China) Limited (Beijing, China)	133.3–200
Dinotefuran	Suspension Concentrate (SC)	Neonicotinoid	20%	Xinbaihu Biotechnology Co. Ltd. (Nantong, China)	666.6–1333.2
Acetamiprid	Emulsifiable Concentrate (EC)	Neonicotinoid	10%	Qingdao Taiyuan Technology Development Co., Ltd. (Qingdao, China)	50–100
Thiacloprid	Suspension Concentrate (SC)	Neonicotinoid	40%	Limin Group Co., Ltd. (Linyi, China)	0.33–0.66
Nitenpyram	Water-Dispersible Granule (WG)	Neonicotinoid	20%	Beijing Huarong Kaiwei Plant Protection Biological Technology Co., Ltd. (Beijing, China)	0.5–1
Bifenthrin	Water-Dispersible Granule (WG)	pyrethroid	4.5%	Qingdao Audis Bio-Tech Co. Ltd. (Qingdao, China)	0.66–1.66
Deltamethrin	Emulsion in Water (EW) Emulsifiable	pyrethroid	2.5%	Bayer (China) Limited (Beijing, China)	20–30
Pyridaben	Emulsifiable Concentrate (EC)	Pyridazine ketone	15%	Yifan Biotechnology Group Co., Ltd. (Taizhou, China)	50–70
Flonicamid	Water dispersible Granule (WG)	Pyridine amide	50%	Shandong Yijia Agrochemical Co., Ltd. (Linyi, China)	140–233.3
Emamectin benzoate	Suspension Concentrate (SC)	Organophosphorus	11.6%	Keagio (Chengdu, China)	17–20

Recommended field concentration (mg L^−1^): recommended by the manufacturer.

**Table 2:** Susceptibility of field populations of *B. tabaci* adults to thirteen insecticides.

Insecticide	N ^a^	Slope (±SE)	LC_50_ (mg L^−1^)	95% FL ^b^	Df ^c^	χ^2^	RR	Resistance Level
Abamectin	352	0.754 (±0.145)	1.479	0.867–3.959	4	2.995	14.79	Medium
Thiamethoxam	370	1.463 (±0.179)	2102.847	1450.667–3733.475	4	10.401	22.98	Medium
Flupyradifurone	415	1.206 (±0.124)	881.005	675.793–1193.961	4	1.282	121.23	very high
Imidacloprid	375	0.868 (±0.155)	9917.911	4381–88744	4	4.227	320.65	very high
Dinotefuran	364	0.511 (±0.147)	991.653	315.736–42,440.436	4	2.779	111.42	very high
Acetamiprid	459	1.422 (±0.154)	243.428	176.061–358.109	5	6.5828	13.85	Medium
Thiacloprid	367	1.027 (±0.156)	2529.384	1663.349–4924.830	4	0.498	157.01	very high
Nitenpyram	400	1.413 (±0.156)	101.232	63.229–187.475	4	8.1131	1.77	low
Bifenthrin	395	1.057 (±0.137)	138.706	56.841–289.531	4	10.401	6.73	low
Deltamethrin	368	1.112 (±0.155)	774.645	544.228–1298.368	4	0.481	74.69	high
Pyridaben	537	0.520 (±0.083)	1147.928	546.299–4024.331	6	1.626	331.29	very high
Flonicamid	368	0.629 (±0.137)	920.145	505.069–3044.893	4	0.656	27.99	Medium
Emamectin benzoate	230	1.743 (±0.532)	7.516	4.709–41.483	2	1.429	2.13	low

N ^a^: total number of adults in bioassay. SE: standard error. LC_50_: concentration of insecticide killing 50% of adults. 95% FL ^b^: 95% fiducial limits. Df ^c^: degree of freedom. χ^2^: chi-square testing linearity of dose–mortality responses. RF: resistance ratio = the LC_50_ value of the field population divided by the LC_50_ value of the baseline.

## Data Availability

The original contributions presented in this study are included in the article. Further inquiries can be directed to the corresponding author.
